# A Multiplatform Metabolomic Approach to the Basis of Antimonial Action and Resistance in *Leishmania infantum*

**DOI:** 10.1371/journal.pone.0130675

**Published:** 2015-07-10

**Authors:** David Rojo, Gisele A. B. Canuto, Emerson A. Castilho-Martins, Marina F. M. Tavares, Coral Barbas, Ángeles López-Gonzálvez, Luis Rivas

**Affiliations:** 1 Centro de Metabolomica y Bioanalisis (CEMBIO), Unidad Asociada Interacciones, Metabolismo y Bioanálisis CSIC-CEU, Facultad de Farmacia, Universidad CEU San Pablo, Campus Monteprincipe, Boadilla del Monte, 28668, Madrid, Spain; 2 Institute of Chemistry, University of São Paulo (USP), Campus São Paulo, São Paulo, 05508–000, São Paulo, Brazil; 3 Colegiado de Medicina, Universidade Federal do Amapá, Macapá, AP, Brazil; 4 Centro de Investigaciones Biológicas (CSIC), Unidad Asociada Interacciones, Metabolismo y Bioanálisis CSIC-CEU, Ramiro de Maeztu 9, 28040, Madrid, Spain; University of Nebraska-Lincoln, UNITED STATES

## Abstract

There is a rising resistance against antimony drugs, the gold-standard for treatment until some years ago. That is a serious problem due to the paucity of drugs in current clinical use. In a research to reveal how these drugs affect the parasite during treatment and to unravel the underlying basis for their resistance, we have employed metabolomics to study treatment in *Leishmania infantum* promastigotes. This was accomplished first through the untargeted analysis of metabolic snapshots of treated and untreated parasites both resistant and responders, utilizing a multiplatform approach to give the widest as possible coverage of the metabolome, and additionally through novel monitoring of the origin of the detected alterations through a ^13^C traceability experiment. Our data stress a multi-target metabolic alteration with treatment, affecting in particular the cell redox system that is essential to cope with detoxification and biosynthetic processes. Additionally, relevant changes were noted in amino acid metabolism. Our results are in agreement with other authors studying other *Leishmania* species.

## Introduction

The term leishmaniasis encompasses the different clinical infections by protozoan species belonging to the genus *Leishmania*, whose importance for human health is only superseded by malaria. Worldwide, leishmaniasis accounts for 10–12 million infected people, with 2 million new cases per year and near 50,000 annual deaths [1]. Nowadays, treatment relies almost exclusively on chemotherapy; reduced to a small number of drugs with an effectiveness increasingly eroded by rising resistance, severe side-effects and unaffordable costs for massive implementation. Additionally, there is a shortfall of new candidates in the pipeline.

Since their introduction 60 years ago, organic pentavalent antimonials have constituted the gold standard for treatment until clinical resistance in Northwest India and Nepal reached an inadmissible rate of 60%, as reported even in patients without any prior treatment [2] leading to their progressive demotion by miltefosine or liposomal amphotericin B, especially for the visceral forms of the disease. Nevertheless, the current use of antimonials is still quite notorious in canine leishmaniasis in combination with allopurinol [3] and concerning human clinics, as monotherapy on some endemic areas of visceral leishmaniasis as well as on mucocutaneous and cutaneous forms of the disease [4], and finally as part of drug combination strategies [5], mostly based on clinical experience, rather than on knowledge of their mechanism of action and resistance and the targets involved. In this sense, a deeper insight into these items will ameliorate the clinical strategies to tackle the disease, in an attempt to avoid partial cross-resistance of antimonials with other drugs as observed in field studies [6–8]. This, together with vector transmission of the resistant phenotype aforementioned [9], and the improved fitness of resistant parasites inside the host [10], foresees a privileged expansion of the antimonial resistant parasites over the susceptible isolates [11].

Among anti *Leishmania* drugs, mechanisms of action of and resistance to antimonials are those with higher complexity. Their lethal mechanism is mostly based on deterioration of the thiol-dependent metabolism of the parasite [12–15], induced by the inhibition of trypanothione reductase and trypanothione depletion after its conjugation with Sb^3+^ [13], with a concomitant global dysfunction of the parasite metabolism and structural functionality [16–20].

The core of resistance to antimonials relies on a two-pronged strategy in *Leishmania*. First, the prevention of intracellular build-up of Sb^3+^, the toxic form of antimonials, by i) down expression of the parasite reductases responsible for the reduction of the administered prodrug, Sb^5+^, into Sb^3+^ [21,22], ii) impairment of the entry of external Sb^3+^ into the parasite through decreased levels of aquaglyceroporin 1 [23], and iii) decreasing the levels of intracellular Sb^3+^ by efflux pumps [8,17,24]. The second set of strategies were aimed to restore the redox power, crippled by the drug, by overexpression of enzymes of polyamine and trypanothione biosynthesis, with increment of intracellular levels of this metabolite and of glutathione, its precursor [25,26].

Furthermore, the final clinical outcome for antimonials encompassed additional factors such as the modulation of the physiology of the macrophage by these drugs [24,27,28], the strength of the immune status of the host [29] and, surprisingly, environmental factors, as the exposure of human population to low concentrations of As^3+^ in drinking water [30], that predisposed for antimonial resistance.

Unbiased “omics” studies of changes associated to the action of and resistance to antimonials have been carried out through genomics [17,31–33] and proteomics [17–20,34–36]. However, among the unbiased “omics” only metabolomics provides a direct snapshot of the final effect of the drug and how it challenges parasite metabolism [37–41]. The importance of this technique in *Leishmania* is highlighted by the utmost importance of the post-transcriptional control of gene expression operative in trypanosomatids compared with higher eukaryotes [42,43]. The potentiality of metabolomics to unravel the mechanism of action of antimonials is supported by previous works on *L*. *donovani* isolates from India [38,41] and on a reference strain of *L*. *infantum* from Spain [40], using for separation of metabolites ZIC-HILIC chromatography or capillary electrophoresis (CE), respectively.

The goal of this work is to test whether a metabolomic platform, gathering different complementary analytical techniques, will improve the metabolite coverage achieved by single separation techniques, applied to the mechanism of action and resistance to Sb^3+^ in *Leishmania infantum*. To achieve this purpose we have used exponential promastigotes from a reference strain of this species and its resulting Sb^3+^ resistant isolate, obtained by growing the parasites *in vitro* under a stepwise increasing concentration of Sb^3+^. Although a full extrapolation of the strategies adopted by the promastigote to cope with the metalloid stress into amastigotes will be unfeasible due to the extensive interstage metabolic rewiring, we surmise that most of the results obtained are shared by both stages; first, Sb^3+^,the oxidation stage of antimony used, is the toxic form of the metalloid for both stages; secondly, for a given strain the trait for antimonial-resistance is not only displayed by both stages of the parasite, but maintained through vector transmission [9,11]; finally, the results obtained point out to bioenergetic and redox metabolism, key processes with identical pathways, regardless of the stage of the life cycle of *Leishmania*. The use of promastigotes with a more active and, presumably, more flexible metabolism than the metacyclic form in a *Leishmani*a species, will unveil the contribution of metabolic pathways unnoticed in metacyclic promastigotes. Additionally, the use of *L*. *infantum* strains may, in some degree, decrease the risk of previous antimonials exposure, as frequently happen with the anthoponotic transmission cycles for *L*. *donovani*. Moreover, the difficulty in growing anexic amastigotes of most *Leishmania* species means that the majority of the studies have to be done on cultured promastigotes cells [44].

## Materials and Methods

### Reagents and solvents

Methanol (LC-MS grade), heptane (GC-MS grade), chloroform (MS grade), acetonitrile (LC-MS grade), isopropanol (LC-MS grade), formic acid (MS grade), pyridine (silylation grade), C18:0 methyl ester and O-methoxyamine hydrochloride were purchased from Sigma-Aldrich (Taufkirchen, Germany); N,O-bis(trimethylsilyl) trifluoroacetamide (BSTFA) plus 1% trimethylchlorosilane (TMCS) was purchased from Pierce Chemical Co (Rockford, IL, USA) and references masses purine and HP-0921 (hexakis-(1H,1H,3Htetrafluoropentoxy)-phosphazene) were from Agilent (atmospheric pressure inlet – time of flight (API-TOF) reference mass solution kit). Milli-Qplus 185 was provided by Millipore (Billerica, MA, USA).

### Sample collection and preparation

*L*. *infantum* promastigotes strain M/CAN/ES/96/BCN150 were kindly provided by Dr. F. J. Carrion (Veterinary School, University of Madrid). Parasites were grown in RPMI-1640 medium supplemented with 10% heat inactivated fetal calf serum at 26°C. BCN150 *L*. *infantum* promastigotes resistant to Sb^3+^ were obtained from the parental strain by stepwise culture under increasing concentration of the drug until growth at 180 μmol × L^-1^ potassium antimony(III) tartrate was achieved. Large-scale culture was carried out in a roller apparatus (Gibco, Cell Culture) with an inoculum of 4 × 10^5^ promastigotes × mL^-1^. Once they reached a mid- exponential phase of growth (8 × 10^6^ promastigotes × mL^-1^) they were transferred into the same volume of fresh medium, and incubated or not with 120 μmol × L^-1^ potassium antimony(III) tartrate (a concentration causing 90% mortality in wild type parasites) for 12h. The BCN150 strain has an IC_50_ = 20.9 μmol × L^-1^ with respect to Sb^3+^. Once harvested, parasites were washed twice with Hanks medium at 4°C and immediately frozen in liquid N_2_ and kept at – 80°C until further processing.

The preparation for ^13^C analysis was done by growing the parasites in RPMI where L-arginine was substituted by ^13^C L-arginine (Sigma-Aldrich—Steinheim, Germany, ^13^C in all carbons) for 14h.

### Investigated Groups

**ST:** susceptible strain treated, **SNT**: susceptible strain non–treated (controls) and **R**: resistant strain to Sb^3+^. Four replicates from each group were used in metabolic fingerprinting analysis.

### LC-MS sample treatment

Samples corresponding to 4 × 10^7^ *L*. *infantum* promastigotes were re-suspended in 200 μL MeOH/H_2_O (4/1, v/v) and disrupted in a TissueLyser LT (Qiagen, Germany) (2 glass balls of 2 mm, 5 min, 50 Hz). Solid parts were removed by centrifugation (centrifuge 5415 R Eppendorf) (15700 × *g*, 4°C, 20 min). The supernatant was collected and filtered through a 0.22 μm nylon filter (National Scientific – 197 Cardiff Valley Road, Rockwood, TN 37854, India) and then directly injected into the equipment.

### CE-MS sample treatment

After disruption of the cellular pellets, as described for LC-MS (liquid chromatography-mass spectrometry), 150 μL of the supernatant were transferred into a new tube and evaporated to dryness in a SpeedVac SPD121P (Thermo Fisher Scientific, Waltham, MA) at 35°C. The metabolite extracts were re-suspended in 150 μL MilliQ water, centrifuged (15700 × *g*, 15 min, 4°C) and injected into the instrument.

### GC-MS sample treatment and derivatization

Pellets corresponding to 4 x 10^7^ *L*. *infantum* promastigotes were re-suspended and lysed by addition of 350 μL of cold (4°C) solution of methanol/chloroform/water (3/1/1, v/v/v), and processed for disruption as described for LC-MS samples. Two hundred μL of the resulting supernatant were transferred into a vial and evaporated to dryness in a SpeedVac SPD121P (Thermo Fisher Scientific, Waltham, MA.) at 35°C. For methoximation, 10 μL of *O*-methoxyamine hydrochloride (15 mg × mL^-1^) in pyridine were added to each GC (gas chromatography) vial and vortexed vigorously (FB 15024, Fisher Scientific, Spain). The vials were incubated in darkness at room temperature for 16 hours. Then, 10 μL of BSTFA with 1% TMCS (v/v) were added and samples were vortexed for 5 min, silylation was carried out for 1 h at 70°C and finally 100 μL of C18:0 methyl ester (10 mg × L^-1^ in heptane) were added as an internal standard and samples were mixed again by vortexing gently. Two blank samples were prepared by the same procedure of extraction and derivatization and analyzed at the beginning and at the end of the sequence.

### Quality Controls

QC samples were prepared by pooling equal volumes of each sample from all groups. The same procedure was followed for the three techniques (before the derivatization step in GC-MS). They were analysed throughout the run to provide a measurement of the stability and performance of the system [45] as well as the reproducibility of the procedure for sample treatment.

### Metabolomics fingerprinting by LC-ESI-QTOF-MS

The LC system consisted of a degasser, two binary pumps and autosampler (1200 series, Agilent); 15 μL of filtered supernatant sample were injected onto a reversed-phase column (Discovery HS C18 150 × 2.1 mm, 3 μm; Supelco) with a guard column (Discovery HS C18 20 × 2.1 mm, 3 μm; Supelco) kept at 40°C. The system was operated at a flow rate of 0.6 mL × min^-1^ with solvent A (0.1% formic acid in water) and B (0.1% formic acid in acetonitrile). The gradient started from 25% B to 95% B in 35 min, followed by 5 min of 95% of B and then returned to starting conditions in 1 min, with a re-equilibration step at 25% B for 9 min (total analysis time 50 minutes). Data were collected in positive ESI (electrospray) mode in separate runs on a QTOF (quadrupole time-of-flight) (Agilent 6520) operated in full scan mode from *m/z* 50 to 1000. The capillary voltage was 3000 V with a scan rate of 1.95 scans per second. The gas temperature was 330°C, the drying gas flow 10.5 L × min^-1^ and the nebulizer set up at 52 psi. The MS-TOF (time-of-flight) parameters were: fragmentor 175 V, skimmer 65 V and octopole radio frequency voltage (OCT RF Vpp) 750 V. During the analysis, two reference masses were used: 121.0509 (C_5_H_4_N_4_) and 922.0098 (C_18_H_18_O_6_N_3_P_3_F_24_). They were continuously infused into the system to allow constant mass correction. Samples were analyzed in a randomized run, during which time they were kept in the LC autosampler at 4°C.

### Metabolomics fingerprinting by CE-ESI-TOF-MS

The equipment consisted of a Capillary Electrophoresis (7100 Agilent) coupled to a TOF Mass Spectrometry (6224 Agilent). The CE mode was controlled by ChemStation software (B.04.03, Agilent) and MS mode by Mass- Hunter Workstation Data Analysis (B.02.01, Agilent). The separation occurred in a fused-silica capillary (Agilent) (total length, 100 cm; internal diameter, 50 μm). It was carried out in normal polarity with a background electrolyte containing 0.8 mol × L^-1^ of formic acid solution in 10% methanol (v/v) at 20°C. New capillaries were pre-conditioned by flushing successively by NaOH 1.0 mol ×L^-1^ MilliQ water and background electrolyte, 30 min each. Before each analysis the capillary was conditioned by flushing of background electrolyte for 5 min. The sheath liquid (6 μL × min^-1^) was methanol/water (1/1, v/v) containing 1.0 mmol × L^-1^ formic acid with two reference masses: 121.0509 (C_5_H_4_N_4_) and 922.0098 (C_18_H_18_O_6_N_3_P_3_F_24_), to allow correction and higher accurate mass in the MS. Samples were hydrodynamically injected at 50 mbar for 50 s and stacked by applying background electrolyte at 100 mbar for 10 s. The separation voltage was 30 kV with 25 mbar of internal pressure and the analyses were carried out in 30 min. The MS parameters were optimized [40] by the study of signal-to-noise ratio of the ion 175.1179 Da (corresponding to L-arginine, previously identified in *Leishmania*); the best analytical conditions were: fragmentor 100 V, skimmer 65 V, octopole 750 V, nebulizer pressure 20 psi, drying gas temperature at 200°C and flow rate 12.0 L x min^-1^. The capillary voltage was 3500 V. Data were acquired in positive Dual-ESI mode with a full scan from *m/z* 80 to 1000 at a rate 1.02 scans per second.

### Metabolomics fingerprinting by GC-EI-IT-MS

GC system (Agilent Technologies 7890A) consisted in an auto sampler (Agilent Technologies 7693) and an inert MSD with Quadrupole (Agilent Technologies 5975). 2 μL of the derivatized sample were injected through a GC-Column DB5-MS (30 m length, 0.25 mm internal diameter, 0.25 μm film 95% dimethylpolysiloxane / 5% diphenylpolysiloxane) with a pre-column (10 m J&W integrated with Agilent 122-5532G). The flow rate of the helium carrier gas was set at 1 mL × min^-1^ and the injector temperature 250°C. The split ratio was 1:10 flow into a Restek 20782 deactivated glass-wool split liner. The temperature gradient was programmed at 60°C (held for 1 min), with a ramping increase rate of 10°C × min^-1^ up to 325°C. Finally it was cooled down for 10 min before the next injection. The total analysis time was 37.5 min. The detector transfer line, filament source and quadrupole temperature were respectively set at 290°C, 230°C and 150°C. The electron ionization (EI) source was placed at 70 eV. The mass spectrometer operated in scan mode over a mass range of *m/z* 50–600 at a rate of 2 spectra per second. Peaks detection and spectra processing were obtained using Agilent ChemStation Software (G1701EA E.02.00.493, Agilent).

### Data treatment

The resulting data files (LC-MS and CE-MS) were cleaned of background noise and unrelated ions by the Molecular Feature Extraction (MFE) tool in the Mass Hunter Qualitative Analysis software (B.04.00, Agilent).

Primary data treatment (filtering and alignment) was performed with Mass Profiler Professional software (B.02.01, Agilent). Features were filtered by selecting the data present for LC-MS and CE-MS in at least 50% of the QCs and in 75% of the samples in 1 of the 2 groups: ST *vs*. SNT and R *vs*. SNT, in order to assess the effects of the treatment, and to investigate the metabolic variations in resistance, respectively. Differences for individual metabolites were evaluated by comparison of these two groups using Mann-Whitney U (where *p* values < 0.05 were considered significant). The accurate masses representing statistical significant differences were searched against the METLIN database and MassTrix (Mass Translation into Pathways), a tool for data annotation. Treatment of GC-MS data was carried out as aforementioned for identification, and further deconvolution was performed. AMDIS (automated mass spectral deconvolution and identification v. 2.69) software was used to identify co-eluted compounds according to their retention index and retention time. Then, alignment and filtering steps (presence in at least 75% of the samples in at least one of the groups were done with Mass Profiler Professional software (B.02.01, Agilent), and statistical analysis for treatment (ST *vs*. SNT) and resistance (R *vs*. SNT) carried out as above. Masses with a *p* value < 0.05 were selected.

### Compound identification

LC-MS analysis: the identity of the compounds selected according to their significance in class separation was further confirmed by LC-MS/MS using a QTOF (model 6520, Agilent). Experiments were repeated with identical chromatographic conditions of the primary analysis (described above). Ions were targeted for collision-induced dissociation (CID) fragmentation on the fly based on the previously determined accurate mass and retention time. Identity was confirmed by comparison of the structure of the proposed compound with the fragments obtained. If available, the resulting accurate mass data and isotopic distributions for the precursor and product ions were studied and compared with spectral data of reference compounds obtained under identical conditions, using MS/MS spectra in public database (METLIN- http://metlin.scripps.edu/) or against commercially available standards. A third way of identity confirmation was to check the accurate mass data and isotopic distributions for the precursor and product ions in MS Fragmentor software (12.01, ACD/Labs).

CE-MS analysis: the significant accurate masses were searched against the public databases METLIN: Metabolite and Tandem MS Database (http://metlin.scripps.edu/) and MassTrix: Mass Translation into Pathways (http://metabolomics.helmholtz-muenchen.de/masstrix2/).

GC-MS analysis: the compound identification was performed by using Fiehn RTL Library and the NIST mass spectra library version 2.0g, with the ChemStation software PBM algorithm (G1701EA E.02.00.493, Agilent).

Finally, the presence of the compounds labelled with ^13^C was confirmed by the analysis of its isotopic pattern distribution in LC-MS and CE-MS. The corresponding comparison between the extracted ions chromatograms/electropherograms were done in order to evaluate the discriminating presence of ^13^C for each compound belonging to the metabolic pathways of L-arginine in *Leishmania*.

## Results and Discussion

**Fig 1:**
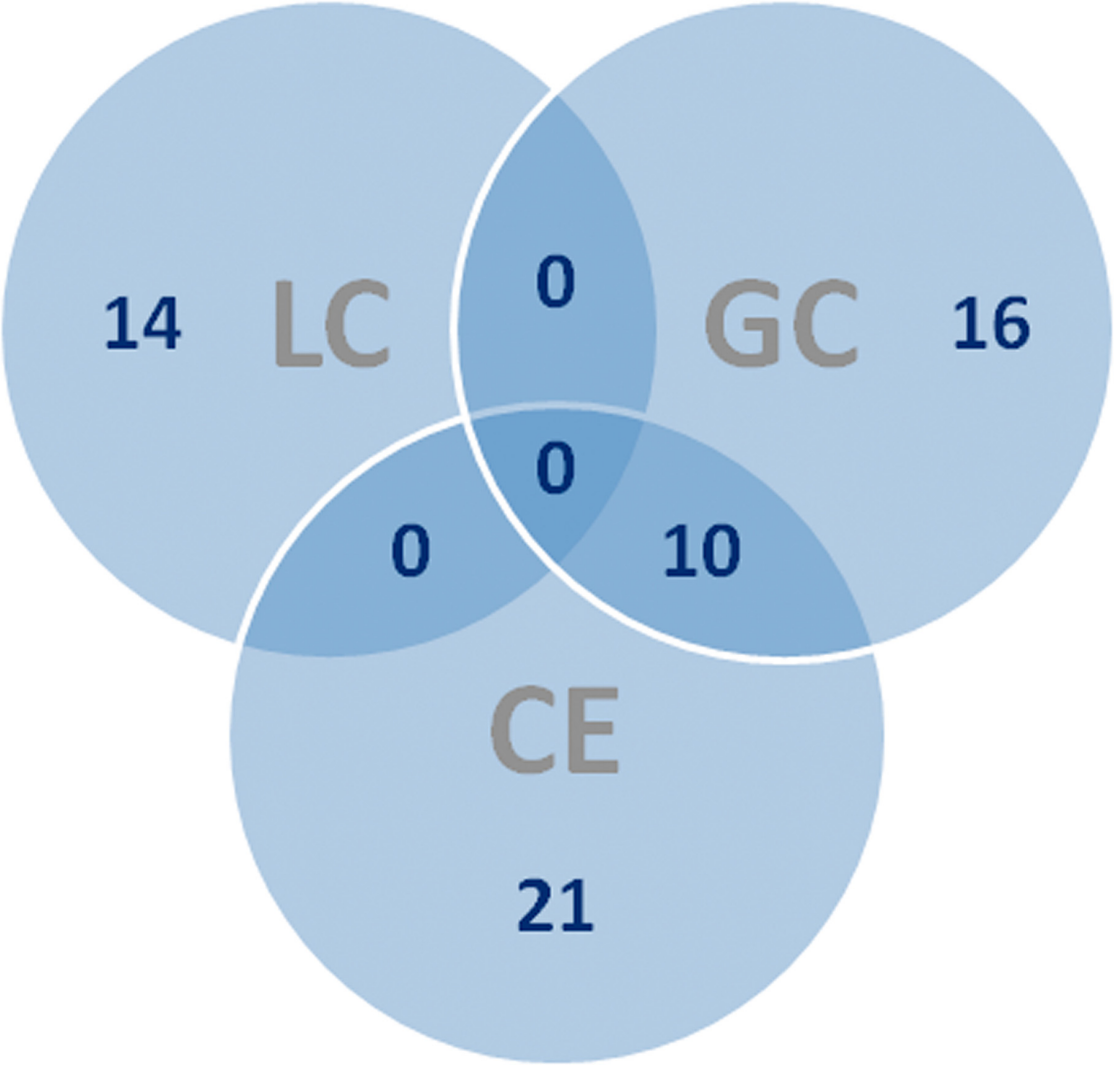
Venn diagram. Venn diagram for the number of compounds identified in each technique.

In this work, we have used three different analytical platforms to cover the widest range of metabolites from *L*. *infantum* promastigotes. Table 1 summarizes the number of features obtained for each step from the corresponding technique, while Fig 1 compiles the number of identified compounds for each technique. It is quite obvious how useful the multiplatform approach is; only 10 out of 61 compounds (16%) were identified by two techniques and none for the three platforms. This means that 84% of metabolites are exclusively identified by one technique; 35% for CE-MS, 26% for GC-MS and 23% from LC-MS/MS.

**Table 1:** Comparison of the different techniques and their respective number of features at the different steps of selection.

		LC-MS		CE-MS		GC-MS	
		ST *vs*.SNT	R *vs*.SNT	ST *vs*.SNT	R *vs*.SNT	ST *vs*.SNT	R *vs*.SNT
**Number of features**	By alignment	7299	7299	1072	1072	89	89
	After statistical treatment	283	278	32	97	23	24
	Identified	14		31		26	

**Fig 2:**
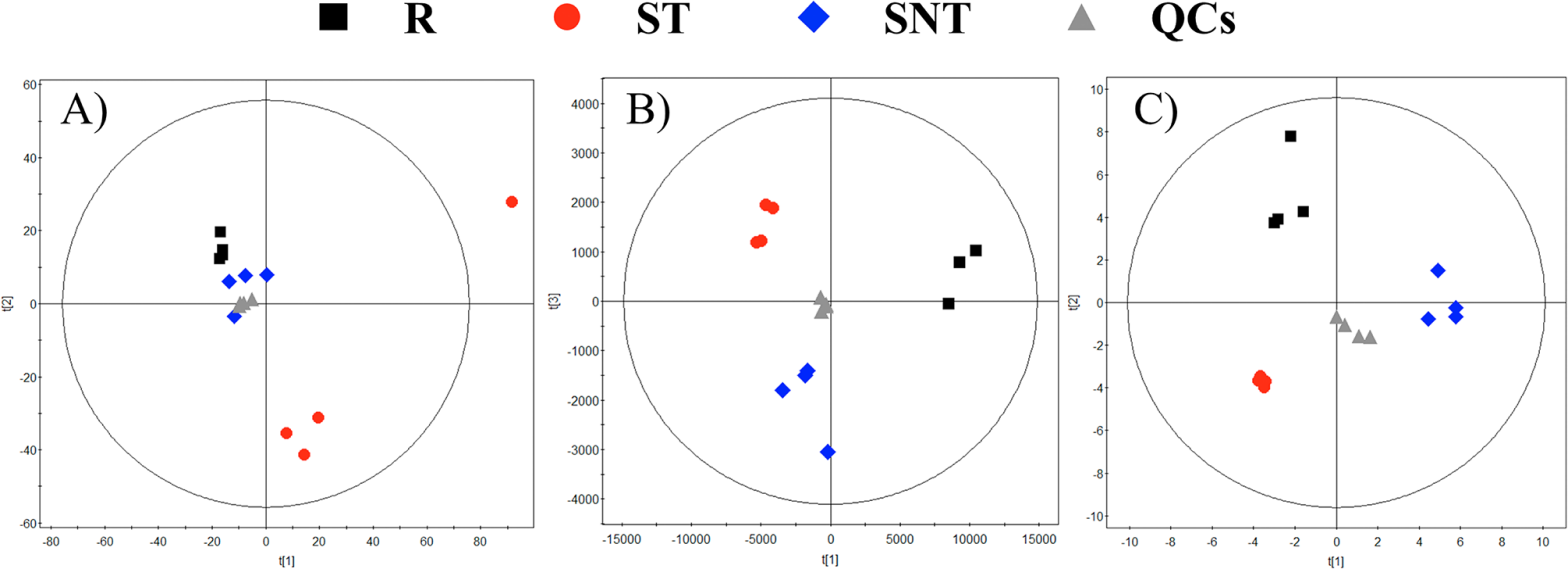
PCA models. PCA models for the whole data set filtered according to their presence in at leasts 50% of the QCs. Panels: A.- LC-MS. 2 components. R^2^ = 0.62. Q^2^ = -0.029. B.- CE-MS. 2 components. R^2^ = 0.872. Q^2^ = 0.694. C.- GC-MS. 2 components. R^2^ = 0.515. Q^2^ = 0.235. Each group is obtained from four samples, except R in CE-MS that consisted of three due to some problems during the sample treatment.

To assess the quality of the analytical techniques, we applied PCA (principal component analysis) models (built by SIMCAP+ software), as represented for LC-MS, CE-MS and GC-MS in Fig 2. The tight clustering of the QCs, located at the center of the plot, evidenced the robustness of the analytical procedure and that separations among the groups obeyed to a real biological differentiation.

The results of putative and confirmed identification by LC-MS/MS, CE-MS and GC-MS are summarized in Table 2, while Table 3 shows the compounds identified by LC-MS/MS and their characteristic fragments.

**Table 2:** Compounds with statistical significance identified and their variation tendency for each of the comparisons.

Compound name	Mass (Da)	Molecular formula	Mass error (ppm)	ST *vs* SNT	*p*-value	R *vs* SNT	*p*-value	CV in QC	Analytical technique	Confirmation	Biochemical nature
(4,8,10-d18:3)Sphingosine	295.2511	C_18_H_33_NO_2_	2			down	< 0.05	6	LC-MS	Id MS/MS	Sphingolipids and spingoid bases
2-Oxo-5-methylthiopentanoic acid	162.0351	C_6_H_10_O_3_S	7	down	< 0.01	down	< 0.01	6	CE-MS	Putative	Organic acids
4-Hydroxy-proline / 5-amino-2-oxopentanoate	131.0582	C_5_H_9_NO_3_	0			up	< 0.05	3	CE-MS	Putative	Amino acids, peptides and conjugates
	131.0582	C_5_H_9_NO_3_		down	< 0.01			7	GC-MS	Identified	Amino acids, peptides and conjugates
4-Oxoproline / L-1-Pyrroline-3-hydroxy-5-carboxylate / 1-Pyrroline-4-hydroxy-2-carboxylate	129.0426	C_5_H_7_NO_3_	1			up	< 0.05	4	CE-MS	Putative	Amino acids, peptides and conjugates
2-Oxoarginine	173.0800	C_6_H_11_N_3_O_3_	0	up	< 0.01	up	< 0.01	12	CE-MS	Putative	Organic acids
Adenosine	267.0968	C_10_H_13_N_5_O_4_		up	< 0.05	down	< 0.05	6	GC-MS	Identified	Purines/pyrimidines and conjugates
ADMA / SDMA	202.1430	C_8_H_18_N_4_O_2_	0	up	< 0.01	up	< 0.05	8	CE-MS	Putative	Amino acids, peptides and conjugates
Alanine / Sarcosine	89.0477	C_3_H_7_NO_2_	6	down	< 0.01	up	< 0.05	4	CE-MS	Putative	Amino acids, peptides and conjugates
Allantoic acid	176.0546	C_4_H_8_N_4_O_4_	10	down	< 0.01	down	< 0.01	8	LC-MS	Id MS/MS	Organic acids
Arginine	174.1117	C_6_H_14_N_4_O_2_	1	down	< 0.05	up	< 0.05	2	CE-MS	Id C13	Amino acids, peptides and conjugates
Asparagine /N-carbamoyl Sarcosine	132.0535	C_4_H_8_N_2_O_3_	5			up	< 0.05	4	CE-MS	Putative	Amino acids, peptides and conjugates
	132.0535	C_4_H_8_N_2_O_3_		down	< 0.01			4	GC-MS	Identified	Amino acids, peptides and conjugates
Aspartic acid	133.0375	C_4_H_7_NO_4_		down	< 0.05			6	GC-MS	Identified	Amino acids, peptides and conjugates
Aspartyl-leucine	246.1216	C_10_H_18_N_2_O_5_	15	up	< 0.05			10	LC-MS	Id MS/MS	Amino acids, peptides and conjugates
Bis(glutathionyl)spermine	780.3622	C_30_H_56_N_10_O_10_S_2_	13			down	< 0.01	17	LC-MS	Id MS/MS	Amino acids, peptides and conjugates
C17 Sphinganine	287.2824	C_17_H_37_NO_2_	4			up	< 0.05	5	LC-MS	Id MS/MS	Sphingolipids and spingoid bases
Cellobiose	342.1162	C_12_H_22_O_11_		down	< 0.05	up	< 0.05	3	GC-MS	Identified	Carbohydrates
Choline	103.0997	C_5_H_13_NO	3	up	< 0.01	up	< 0.05	3	CE-MS	Putative	Amines
Citrulline	175.0957	C_6_H_13_N_3_O_3_	0	down	< 0.01	down	< 0.05	5	CE-MS	Id C13	Amino acids, peptides and conjugates
Cystathionine	222.0674	C_7_H_14_N_2_O_4_S	2	up	< 0.05	up	< 0.01	0	CE-MS	Putative	Amino acids, peptides and conjugates
Ergosterol	396.3392	C_28_H_44_O		up	< 0.01	down	< 0.05	1	GC-MS	Identified	Sterol and prenol lipids
Glucose-6-phosphate	260.0297	C_6_H_13_O_9_P		down	< 0.05	down	< 0.05	6	GC-MS	Identified	Carbohydrates
Glutamate / Isoglutamate / 2- Oxo-4 hydroxy-5-aminovalerate / L4-Hidroxy Glutamate semialdehyde	147.0532	C_5_H_9_NO_4_	0	down	< 0.01	up	< 0.05	15	CE-MS	Putative	Amino acids, peptides and conjugates
Glutamine	146.0691	C_5_H_10_N_2_O_3_		down	< 0.05	down	< 0.05	6	GC-MS	Identified	Amino acids, peptides and conjugates
Glycerophosphocholine	257.1028	C_8_H_20_NO_6_P	1	up	< 0.01			27	LC-MS	Id MS/MS	Glycerophospholipids
Guanine	151.0494	C_5_H_5_N_5_O	0			up	< 0.01	12	CE-MS	Putative	Purines/pyrimidines and conjugates
Histidine	155.0695	C_6_H_9_N_3_O_2_	0			up	< 0.01	6	CE-MS	Putative	Amino acids, peptides and conjugates
	155.0695	C_6_H_9_N_3_O_2_		up	< 0.05			6	GC-MS	Identified	Amino acids, peptides and conjugates
Hypoxanthine	136.0385	C_5_H_4_N_4_O	0	down	< 0.05			5	CE-MS	Putative	Purines/pyrimidines and conjugates
	136.0385	C_5_H_4_N_4_O		up	< 0.01	up	< 0.01	4	GC-MS	Identified	Purines/pyrimidines and conjugates
Imidazole lactate	156.0535	C_6_H_8_N_2_O_3_	0	down	< 0.01	up	< 0.01	5	CE-MS	Putative	Organic acids
Lauric acid	200.1776	C_12_H_24_O_2_	1	up	< 0.05			5	LC-MS	Id MS/MS	Fatty acyls
Lauric Acid ethyl ester	228.2089	C_14_H_28_O_2_	1			down	< 0.05	5	LC-MS	Id MS/MS	Fatty acyls
Leucine	131.0946	C_6_H_13_NO_2_	0	down	< 0.05	down	< 0.05	6	CE-MS	Putative	Amino acids, peptides and conjugates
	131.0946	C_6_H_13_NO_2_		down	< 0.05			2	GC-MS	Identified	Amino acids, peptides and conjugates
Leucyl-Alanine	202.1317	C_9_H_18_N_2_O_3_	0			up	< 0.05	7	CE-MS	Putative	Amino acids, peptides and conjugates
Linoleic acid	280.2402	C_18_H_32_O_2_		up	< 0.01	up	< 0.01	3	GC-MS	Identified	Fatty acyls
Linolenic Acid	278.2246	C_18_H_30_O_2_	0	up	< 0.05	down	< 0.01	3	LC-MS	Id MS/MS	Fatty acyls
Lysine	146.1055	C_6_H_14_N_2_O_3_	0			up	< 0.05	1	CE-MS	Putative	Amino acids, peptides and conjugates
	146.1055	C_6_H_14_N_2_O_3_		up	< 0.01	up	< 0.01	5	GC-MS	Identified	Amino acids, peptides and conjugates
Malic acid	134.0215	C_4_H_6_O_5_		down	< 0.05	down	< 0.01	4	GC-MS	Identified	Organic acids
Methylhistidine	169.0851	C_7_H_11_N_3_O_2_	0			up	< 0.05	13	CE-MS	Putative	Amino acids, peptides and conjugates
Myo-inositol	180.0632	C_6_H_12_O_6_		down	< 0.01			4	GC-MS	Identified	Carbohydrates
Myristic acid	228.2089	C_14_H_28_O_2_		down	< 0.05	up	< 0.05	5	GC-MS	Identified	Fatty acyls
Myristic Acid ethyl ester	256.2402	C_16_H_32_O_2_	1	up	< 0.05			10	LC-MS	Id MS/MS	Fatty acyls
N-Acetylaminobutanal/ Pipecolic acid	129.0790	C_6_H_11_NO_2_	0			up	< 0.05	20	CE-MS	Putative	Ketones and aldehydes
N-Acetyl-lysine	188.1161	C_8_H_16_N_2_O_3_	1			up	< 0.05	12	CE-MS	Putative	Amino acids, peptides and conjugates
Norleucine	131.0946	C_6_H_13_NO_2_	19			down	< 0.05	8	LC-MS	Id MS/MS	Amino acids, peptides and conjugates
Ornithine	132.0899	C_5_H_12_N_2_O_2_	0	down	<0.05	up	< 0.05	3	CE-MS	Id C13	Amino acids, peptides and conjugates
	132.0899	C_5_H_12_N_2_O_2_		down	< 0.05			3	GC-MS	Identified	Amino acids, peptides and conjugates
Palmitic Acid ethyl ester	284.2715	C_18_H_36_O_2_	3	up	< 0.01			11	LC-MS	Id MS/MS	Fatty acyls
Phosphocholine	183.0660	C_5_H_14_NO_4_P	2	up	0.011	down	< 0.01	28	LC-MS	Id MS/MS	Amines
Phytosphingosine	317.2930	C_18_H_39_NO_3_	3	up	0.012			8	LC-MS	Id MS/MS	Sphingolipids and spingoid bases
Proline	115.0633	C_5_H_9_NO_2_	1			up	< 0.05	11	CE-MS	Putative	Amino acids, peptides and conjugates
Putrescine	88.1000	C_4_H_12_N_2_	6	down	< 0.01			4	CE-MS	Putative	Amines
	88.1000	C_4_H_12_N_2_				up	< 0.01	5	GC-MS	Identified	Amines
Ribitol / Arabitol / Xylitol	152.0685	C_5_H_12_O_5_		up	< 0.01	up	< 0.05	4	GC-MS	Identified	Carbohydrates
Ribose / Lyxose	150.0528	C_5_H_10_O_5_		down	< 0.05	down	< 0.05	4	GC-MS	Identified	Carbohydrates
Ribose-5-phosphate	228.0046	C_5_H_9_O_8_P		up	< 0.01	up	< 0.01	7	GC-MS	Identified	Carbohydrates
S-Adenosylhomocysteine	384.1216	C_14_H_20_N_6_O_5_S	0			up	< 0.05	24	CE-MS	Putative	Purines/pyrimidines and conjugates
S-Adenosylmethionine	398.1372	C_15_H_22_N_6_O_5_S	2	down	< 0.05	up	< 0.05	5	CE-MS	Putative	Purines/pyrimidines and conjugates
Serine	105.0426	C_3_H_7_NO_3_		down	< 0.01	down	< 0.01	3	GC-MS	Identified	Amino acids, peptides and conjugates
Sorbitol / Mannitol	182.0790	C_6_H_14_O_6_		down	< 0.01			3	GC-MS	Identified	Carbohydrates
Threonine	119.0582	C_4_H_9_NO_3_		up	< 0.01			6	GC-MS	Identified	Amino acids, peptides and conjugates
Threonine / Homoserine	119.0582	C_4_H_9_NO_3_	0			up	< 0.05	14	CE-MS	Putative	Amino acids, peptides and conjugates
Trypanothione disulfide	721.2887	C_27_H_47_N_9_O_10_S_2_	0	down	< 0.01	up	< 0.05	1	CE-MS	Putative	Amino acids, peptides and conjugates
Tyrosine	181.0739	C_9_H_11_NO_3_	0			up	< 0.05	4	CE-MS	Putative	Amino acids, peptides and conjugates
	181.0739	C_9_H_11_NO_3_				up	< 0.05	NF	GC-MS	Identified	Amino acids, peptides and conjugates
Valine / Betaine / 5-Aminopentanoate	117.0790	C_5_H_11_NO_2_	1	down	< 0.01	up	< 0.05	7	CE-MS	Putative	Amino acids, peptides and conjugates
Xanthine	152.0334	C_5_H_4_N_4_O_2_				up	< 0.05	2	GC-MS	Identified	Purines/pyrimidines and conjugates

**Table 3:** Compounds identified by LC-MS/MS and their characteristic fragments.

Compound name	Mass (Da)	Molecular formula	Mass error (ppm)	Fragments
(4,8,10-d18:3) Sphingosine	295.2511	C_18_H_33_NO_2_	2	57.07, 81.07, 95.08, 121.10, 162.10, 204.11, 222.12, 279.23
Allantoic acid	176.0546	C_4_H_8_N_4_O_4_	10	43.06, 60.06, 70.06, 71.05, 116.07, 130.09, 134.08, 175.19, 176.10, 177.10
Aspartyl-leucine	246.1216	C_10_H_18_N_2_O_5_	15	70.03, 86.09, 132.10, 141.10, 155.11, 201.12, 247.13
Bis(glutathionyl)spermine	780.3622	C_30_H_56_N_10_O_10_S_2_	13	70.06, 72.08, 86.09, 104.10, 143.03, 158.96, 184.07, 522.75
C17 Sphinganine	287.2824	C_17_H_37_NO_2_	4	69.07, 90.05, 121.10, 164.87, 194.98, 196.97, 227.19, 254.25, 272.25
Glycerophosphocholine	257.1028	C_8_H_20_NO_6_P	1	60.08, 86.09, 104.11, 104.18, 105.11, 124.99, 184.07, 258.11
Lauric acid	200.1776	C_12_H_24_O_2_	1	29.04, 41.04, 43.05, 55.05, 57.07, 69.07, 71.08, 89.06, 103.07, 117.09, 131.10, 151.86, 172.86, 183.85, 201.12
Lauric Acid ethyl ester	228.2089	C_14_H_28_O_2_	1	43.05, 57.07, 71.09, 89.06, 103.07, 117.09, 229.22
Linolenic Acid	278.2246	C_18_H_30_O_2_	0	55.06, 67.06, 81.07, 95.09, 109.10, 123.12, 137.13, 173.13, 279.23
Myristic Acid ethyl ester	256.2402	C_16_H_32_O_2_	1	43.05, 57.07, 71.09, 89.06, 103.07, 117.09, 257.25
Norleucine	131.0946	C_6_H_13_NO_2_	19	30.03, 41.04, 42.04, 43.06, 44.05, 57.07, 69.07, 86.09, 130.16, 131.16, 132.09
Palmitic Acid ethyl ester	284.2715	C_18_H_36_O_2_	3	41.04, 43.06, 55.06, 57.07, 69.07, 71.09, 85.10, 89.06, 95.09, 103.08, 117.09, 135.12, 149.13, 173.15, 201.19, 229.21
Phosphocholine	183.0660	C_5_H_14_NO_4_P	2	45.04, 60.08, 86.1, 98.98, 124.99, 184.07
Phytosphingosine	317.2930	C_18_H_39_NO_3_	3	41.04, 55.02, 71.05, 95.05, 113.06, 135.11, 171.10, 219.17, 252.26, 282.27, 300.28, 318.30

**Fig 3:**
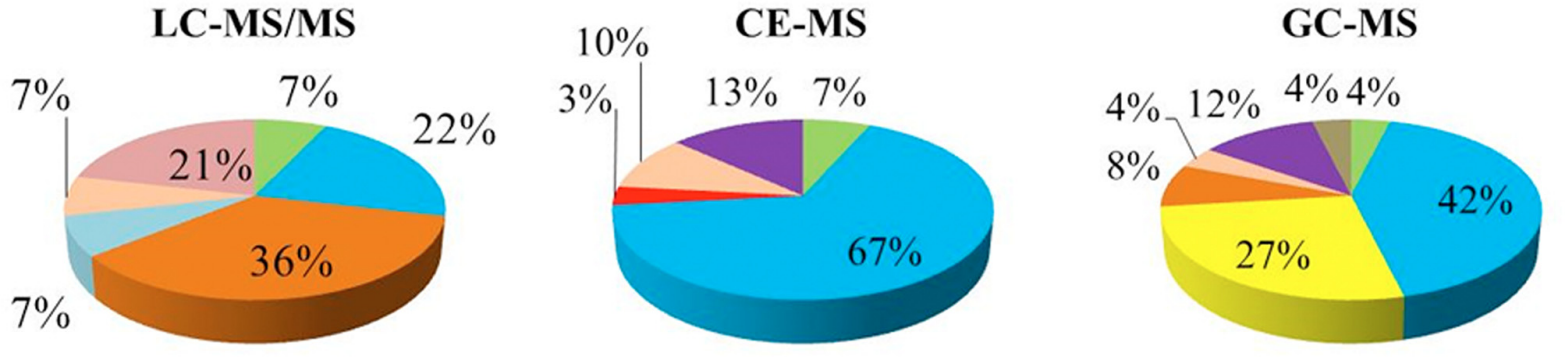
Biochemical classification. Biochemical classification of the identified compounds per each technique expressed as percentage. Green: amines. Cyan: amino acids, peptides and conjugates. Yellow: carbohydrates. Orange: fatty acids. Light blue: glycerophospholipids. Red: ketones and aldehydes. Ochre: organic acids. Purple: purines, pyrimidines and conjugates. Pink: sphingolipids and spingoid bases. Brown: sterol and prenol lipids.

According to biochemical criteria, amino acids, peptides and conjugates were the largest category of identified compounds (Fig 3) accounting for at least 22% of the total obtained for the different techniques, followed by organic acids and amines. Nevertheless, the most important differences came from the comparison among techniques. In CE-MS the amino acids, peptides and conjugates were the largest group (67%) followed by purines, pyrimidines and their conjugates (13%) and by organic acids (10%). In GC-MS, amino acids (42%), carbohydrates (27%) and purines and pyrimidines (12%) were identified as the main groups; most importantly, the carbohydrates and the sterol and prenylated lipids were exclusively identified in this analytical platform. Finally, in LC-MS/MS, the most populated category corresponded to fatty acids (36%), followed by amino acids (22%), and sphingolipids and spingoid bases (21%). Fatty acids, sphingolipids and glycerophospholipids were uniquely identified by this technique.

**Fig 4:**
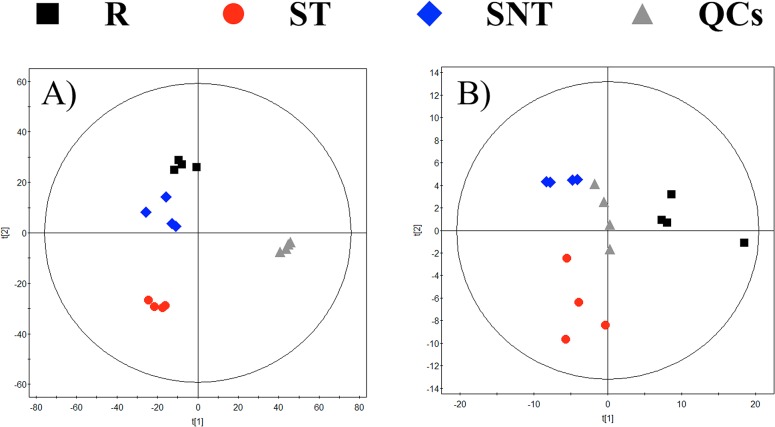
PCA models for ^13^C. PCA models for the whole data set filtered according to their presence in at leasts 50% of the QCs for the ^13^C arginine experiment. Panels: A LC-MS. 3 components. R^2^ = 0.428. Q^2^ = 0.107. B.- CE-MS. 2 components. R^2^ = 0.577. Q^2^ = 0.424.

**Fig 5:**
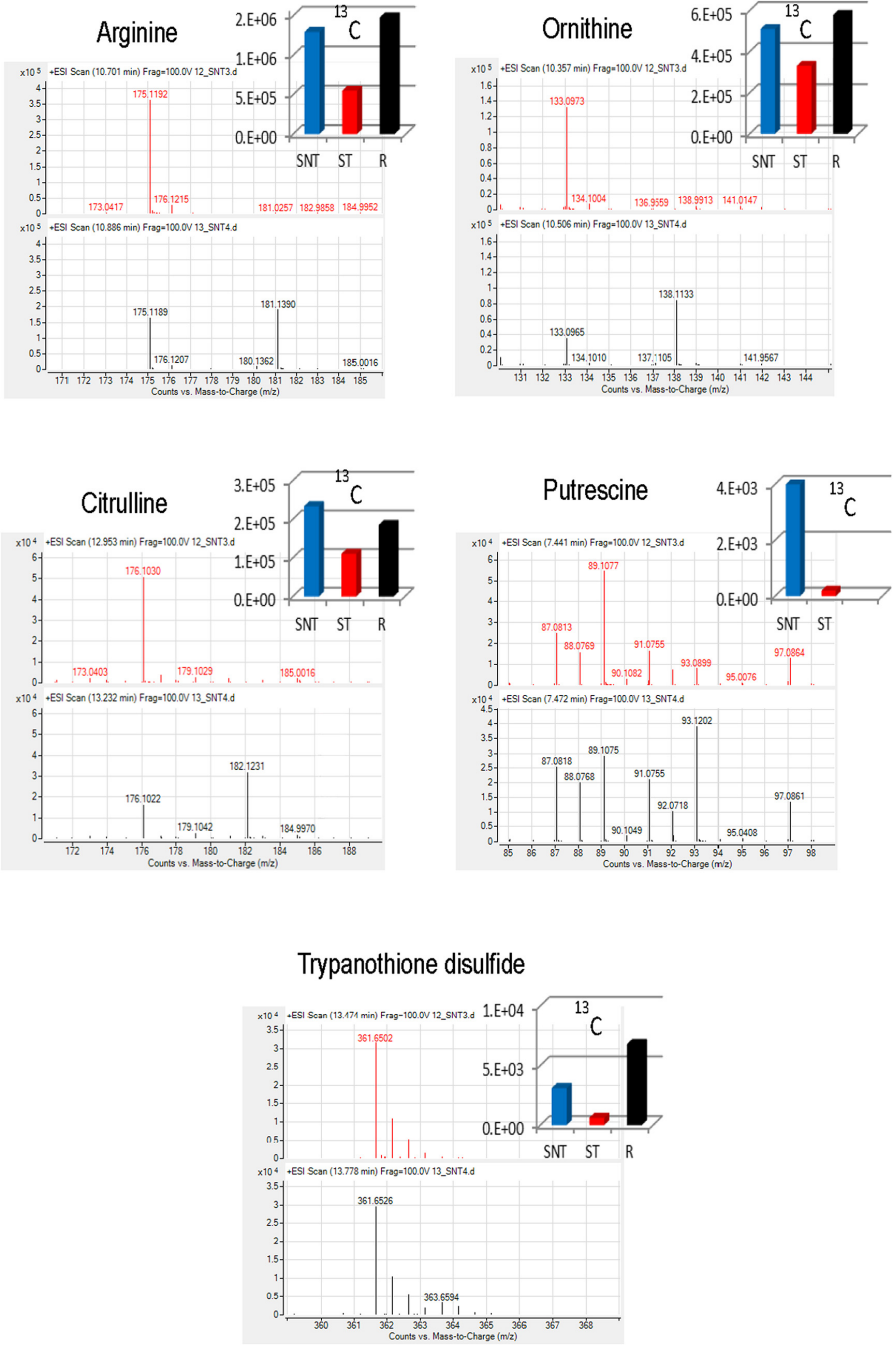
^13^C labeled compound. ^13^C labeled compound and their average abundance per group for the ^13^C-L-arginine experiment. Spectra from ^12^C and ^13^C samples appeared in red and black, respectively. Average abundance for each isotope appeared in the histogram.

In order to pinpoint the metabolic origin of those compounds with statistically significant differences, the same experiment was repeated in the presence of the ^13^C isotopic form of L-arginine uniformly labelled in the culture medium. Once metabolomic analysis by LC-MS and CE-MS was carried out, multivariate analysis of the results showed a significant separation among the groups (Fig 4); this can be considered an additional biological validation of the first experiment. The list of confirmed compounds with ^13^C and their average abundance per group is shown in Fig 5.

Their most important effect consists in the deterioration of the thiol-dependent redox system of the parasite, leading to an increasing vulnerability against oxidative stress from its own metabolism, aggravated by the mitochondrial dysfunction caused by Sb^3+^ [46], and from the leishmanicidal mechanisms of the macrophage as host cell for *Leishmania*. This shortage of glutathione-derived metabolites may also affect glyoxal detoxification or ascorbate reduction pathways, as well as the levels of glutathionylated proteins [47,48].

The metabolic retooling of *Leishmania* to cope the metalloid stress can be gathered under major metabolic groups.

**Fig 6:**
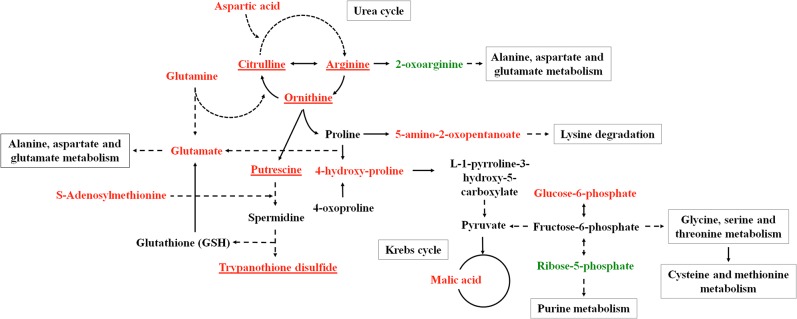
*Leishmania infantum* altered metabolic pathways in ST *vs* SNT. *Leishmania infantum* metabolic pathways with the higher divergence among the groups of parasites studied (ST *vs* SNT strains). Variation of the metabolites inside the metabolic pathways are represented in green (increase), red (decrease) or black (lack of statistical significance). Those that incorporates ^13^C appeared underlined.

**Fig 7:**
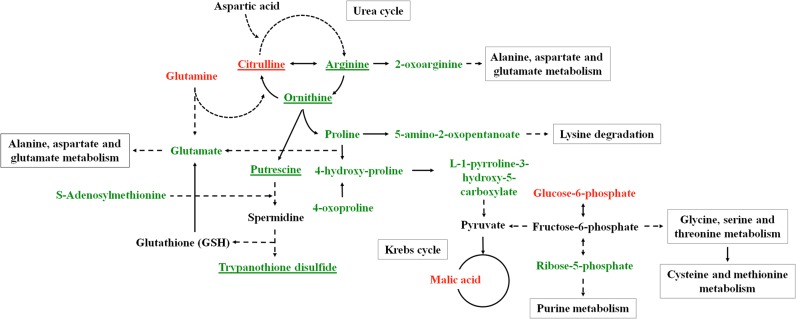
*Leishmania infantum* altered metabolic pathways in R *vs* SNT. *Leishmania infantum* metabolic pathways with the higher divergence among the groups of parasites studied (R *vs* SNT strains). Variation of the metabolites inside the metabolic pathways are represented in green (increase), red (decrease) or black (lack of statistical significance). Those that incorporates ^13^C appeared underlined.

On one hand, when compared with untreated parasites (SNT), Sb^3+^ treated promastigotes (ST) underwent a depletion of metabolites (ornithine, putrescine, and trypanothione) across the arginine → polyamine → trypanothione axis, likely impairing its ability to control the homeostasis of its thiol-dependent intracellular redox environment [49]. It was previously proved that antimonial treatment increased the need of biosynthesis of trypanothione lost by its conjugation to Sb^3+^ to detoxify the parasite [13]. The maintenance of arginine levels, as initial biosynthetic precursor for polyamines, ruled out defects in its uptake, a highly regulated system, [50] as the origin for the levels of downstream metabolites of this pathway. Preliminary experiments of fluxomics, using ^13^C-arginine led to a significant incorporation of ^13^C label into ornithine, citrulline, putrescine and trypanothione disulfide (Fig 5). Furthermore, the level of thiols appeared consistently increased in antimonial resistant parasites. In this regard, the higher level of histidine, the biosynthetic precursor of ovothiol (N(1)-methyl-4-mercaptohistidine), may work as a compensation mechanism to recover an adequate redox power of the parasite. Unfortunately none significant variation for ovothiol was experimentally detected. In fact, glutathione-dependent thiols, but not ovothiol, increased in *L*. *donovani* antimonial resistant isolates [51]. The variation of metabolites inside this pathway with their corresponding trend for comparison of SNT with ST or R parasites is annotated in Figs 6 and 7.

A second observation in Sb^3+^ treated parasites concerns the decrease of metabolites used as bioenergetic substrates by the parasite trough glycolysis and oxidative phosphorylation, the latter being the main contributor [52]. Treated parasites (ST) showed decreased levels of i) glutamate and glutamine, two amino acids entering the TCA (tricarboxylic acid cycle) via α-ketoglutarate; ii) asparagine and aspartate that do via oxaloacetate, and iii) malate, a direct intermediate of the TCA. Furthermore, aspartate and glutamate, together with malate and α-ketoglutarate are involved in the electron transport from the cytoplasm into the mitochondria. In all, this discloses a dysfunctional Krebs cycle. Proline, another important amino acid as bioenergetic source in *Leishmania*, did not undergo a noticeable decrease.

A recent work on the metabolic basis of the stringent response of the amastigotes inside macrophages, disclosed a major role of mitochondrial metabolism in this stage [53], hence of a higher susceptibility of this form to antimonials [54], as supported by the decrease of the mitochondrial electrochemical potential in Sb^3+^ treated *L*. *donovani* amastigotes [55]. Furthermore, in ST parasites, the levels of two glycolytic intermediates, glucose-6-phosphate and dihydroxyacetone phosphate decreased, making unlikely a higher contribution of glycolysis to compensate for the deficiency in oxidative phosphorylation, as observed for other drugs [56].

If the increase of ribose-5-phosphate is indicative of a higher levels of other intermediates of the pentose phosphate pathway, a higher generation of NADPH, involved in the defense against the oxidative stress created by Sb^3+^, would be inferred [57].

None of the aforementioned metabolic shortages extend into nucleic acid bases, in fact in ST parasites the level of adenosine is even increased. It must to be kept in mind that purinic bases were acquired by savage mechanism; and that *Leishmania* is endowed both with a redundant and interwoven purine and pyrimidine metabolism with an easy interconversion among the molecular species, as well as with transporters with partial overlapping specificities for these bases [58,59].

The analysis of the metabolome for ST parasites suggests potential modification of their membrane composition and architecture. Concerning the lipid bilayer, antimonial treatment lead to a decrease in the ergosterol level, the main sterol in the plasma membrane; whether this is related to the concomitant decrease of leucine, ultimately responsible for ergosterol biosynthesis as its carbon skeleton is incorporated into the mevalonate pathway [60] will require further experiments. Secondly, *myo*-inositol also appeared decreased; 5–10% of the total *Leishmania* lipid is inositol phosphorylceramide [61], and *myo*-inositol the glycosidic moiety of the glycosylphosphatidylinositol anchor that tethers the main components of the plasma membrane to the phospholipid bilayer, such as lipophosphoglycan, Gp63, Gp46 or glycosylinositol phospholipids, with important roles in virulence and survival of the parasite [62]. The decrease in myristate may also impair the N-myristoylation of proteins, a modification required for the functionality of some proteins involved in membrane traffic and signaling [63]. Finally, the increase in choline and specially in glycerophosphocholine points towards an enhanced degradation of phospholipids by phospholipases. If so, an increased fatty acid availability is presumed, which may compensate as energetic fuel of the TCA activity, impaired by the general amino acid shortage. Likewise, concurrent to this membrane remodeling, alanine deficiency, one of the main osmolytes for *Leishmania*, will decrease the capacity of the parasite to cope with osmotic stress [64], which presumably will have an especial relevance for the survival inside the gut of the invertebrate host.

The changes in the metabolome of the resistant parasites showed an opposite trend to ST parasites: R parasites either maintained or increased the pools for metabolites that underwent a decrease in ST, in agreement with the increase in fitness in *L*. *donovani* antimonial resistant parasites [65]. Since macromolecular synthesis is tightly regulated, we infer that most of the changes observed can be interpreted under a metabolic perspective, in agreement with the high percentage of metabolic proteins with significant variation in their expression in antimonials resistance in *L*. *infantum* [17].

Metabolites allotted to arginine-polyamine-trypanothione pathway increased in R parasites over the untreated parasites (SNT) (Fig 7). This encompasses arginine, as the precursor at the top of the pathway, as well as ornithine, a metabolic intermediate, and trypanothione, the final product. In this trend, even the levels of citrulline and 2-oxoarginine, as beacons for minor diverting metabolism of arginine, also appeared decreased in R parasites.

In the same way we may interpret the rise of S-adenosyl methionine, required for spermidine biosynthesis and for the transulfuration in the biosynthesis of cysteine. Interestingly, the S-adenosylmethionine synthase was identified in proteomics of *L*. *panamensis* resistant to Sb^3+^ [36]. In other works overexpression of this enzyme leads to an increase in cysteine [66]. As this was not observed in our results, it may be likely due to a major allocation of this amino acid into the trypanothione biosynthesis. Moreover the levels of glutamate are increased; aside from its role inside the TCA cycle, is one of the amino acids involved in glutathione biosynthesis. Glycine and serine, linked through the triad glycine-serine-cysteine to glutathione biosynthesis, appeared unchanged or decreased. Due to the inconversion for these three amino acids [67], the demand of cysteine will be covered by a higher participation of trans-sulfuration pathway over *de novo* biosynthesis [68].

Similar to ST group, a decrease in glucose-6-phosphate and increase in ribose 5-phosphate were observed in R parasites However, R parasites showed a recovery of dihydroxyacetone phosphate level, a glycolytic metabolite located downstream glycolysis, so despite the decrease in glucose-6-phosphate, levels for glycolytic metabolites appeared to be enhanced in these parasites. In fact, for clinical- and *in vitro*-raised antimonial resistant *Leishmania* isolates from clinical or laboratory origins, higher levels of glycolytic and TCA cycle were disclose by proteomic studies [17,19]. In tune with this, R parasites showed an increment of proline and glutamate, both precursors for TCA intermediates, but the decrease in malate clashes against this scheme; its distribution among glycosome, mitochondria and cytoplasm may blur local variations, we lack a satisfactory explanation to account for this decrease.

Opposite to ST parasites, the signs for architectural remodeling of the membrane are subtler than for R parasites, as for these parasites glycerophosphocholine and myoinositol levels were similar to SNT and increased for myristic acid. Whereas in ST overall PUFA decreased, in R parasites linoleic showed the opposite trends. Nevertheless, the most remarkable changes in membrane components for resistant parasites are sphingolipid and sphingoid bases, C_17_-sphinganine increased whereas 4,8,12 d:18:3 sphingosine decreased. As in *Leishmania* myristoyl-CoA is preferred over palmitoyl-CoA for conjugation with serine [69], from a biosynthesis perspective a predominance of C_16_ over C_17_, thus the change in sphingoid bases will likely be acquired from scavenging from the serum of the medium.

## Conclusions

We have demonstrated that Sb^3+^ mechanism of action and resistance is multifactorial, in agreement to other authors. The combination of the different systems involved gives rise to a heterogeneous pattern, where gene amplification, protein levels, and eventually the metabolome, will change according to the isolate, the species and the pathway to acquire resistance. In any case the metabolome fingerprinting strongly supports redox and bioenergetic metabolism as the axis for Sb^3+^ response. How this can be connected to the large percentage of proteins that underwent variation, is jeopardized as many of these were described as hypothetical proteins [17]. From our point of view, we have provided some valuable clues to solve part of this important puzzle in order to curtail parasite strategies aimed both to mitigate drug effects and to develop resistance. For these two purposes metabolomics may provide a strong support.
